# Clinical outcomes of left bundle branch pacing compared to right ventricular apical pacing in patients with atrioventricular block

**DOI:** 10.1002/clc.23513

**Published:** 2021-03-11

**Authors:** Shigeng Zhang, Junfang Guo, Aibin Tao, Baowei Zhang, Zhonghua Bao, Guohui Zhang

**Affiliations:** ^1^ Department of Cardiology, the First People's Hospital of ZhenJiang Jiangsu University ZhenJiang JiangSu China

**Keywords:** left bundle branch pacing, left ventricular ejection fraction, paced QRS duration, right ventricular apical pacing

## Abstract

**Background:**

Left bundle branch pacing (LBBP) can produce near normalization of QRS duration. This has recently emerged as alternative technique to right ventricular pacing and His bundle pacing.

**Hypothesis:**

The purpose of this study is to evaluate clinical outcomes of LBBP compared to right ventricular apical pacing (RVAP).

**Methods:**

A total of 70 AVB patients with indications for ventricular pacing were retrospectively studied. LBBP was attempted in 33 patients, classified as LBBP group. The other patients were classified as RVAP group. Pacing parameters, electrocardiogram and echocardiogram characteristics, heart failure hospitalization (HFH), and atrial fibrillation (AF) were evaluated perioperatively and at follow‐ups. Patients were followed in the device clinic for a minimum of 12 months and up to 24 months at a 3–6 monthly interval.

**Results:**

LBBP was successful in 29 of 33(87.9%) patients while all 37 of the remaining patients successfully underwent RVAP. Paced QRS duration was significantly narrower in the LBBP group compare to RVAP(110.75 ± 6.77 ms vs. 154.29 ± 6.96 ms, *p* = .000) at implantation, and the difference persisted during follow‐ups. Pacing thresholds (at implantation: 0.68 ± 0.22 V in the LBBP group and 0.73 ± 0.23 V in the RVAP group, *p* = .620) remained low and stable during follow‐ups. The cardiac function in the LBBP group remained stable during follow‐ups (LVEF%:55.08 ± 4.32 pre‐operation and 54.17 ± 4.34 at the end of follow‐up, *p* = .609), and better than RVAP group (LVEF%: 54.17 ± 4.34 vs. 50.14 ± 2.14, *p* = .005). Less HFH was observed in the LBBP group (2/29,6.89%) compared to RVAP group (10/37,27.03%).

**Conclusions:**

The present investigation demonstrates the safety and feasibility of LBBP that produces narrower paced QRS duration than RVAP. LBBP is associated with reduction in the occurrence of pacing‐induced left ventricular dysfunction and HFH compared to RVAP in patients requiring permanent pacemakers.

## 1 INTRODUCTION

For decades, permanent cardiac pacing has been an effective treatment for patients with sick sinus syndrome (SSS) or high‐degree atrio‐ventricular (AV) block. As a conventional pacing strategy, right ventricular apical pacing (RVAP) is easily accessible, stable, and well tolerated.^1^ However, multiple studies have shown that RVAP may lead to pacing‐induced cardiomyopathy (PiCMP) and heart failure (HF), is associated with atrial fibrillation (AF), heart failure hospitalization (HFH), and mortality.^2^, ^3^ The most important reasons are interventricular dyssynchrony and burden of right ventricular (RV) pacing.^4^

Recognition of the deleterious effect of RVAP pacing has led to a continued search for alternate pacing sites, such as RV mid‐septal or outflow tract pacing. But recent studies show that they did not offer any benefits in terms of clinical outcomes over apical lead position.^4^, ^5^, ^6^ In 2000, the pioneering investigation of permanent His bundle pacing (HBP) was first described by Deshmukh et al. in a small series of patients with AF and dilated cardiomyopathy.^7^ Since then, the feasibility and safety of permanent HBP has been demonstrated by several investigators, and HBP is associated with reduction in the combined endpoint of death and HFH compared to RV pacing.^8^, ^9^, ^10^ Although HBP is a physiological alternative to RV pacing, it has not become mainstream therapy, owing to technical challenges and higher and unstable pacing thresholds. In addition, there are longer implantation time, lower R wave amplitude, higher pacing lead revision rate.^8^, ^11^

**FIGURE 1:**
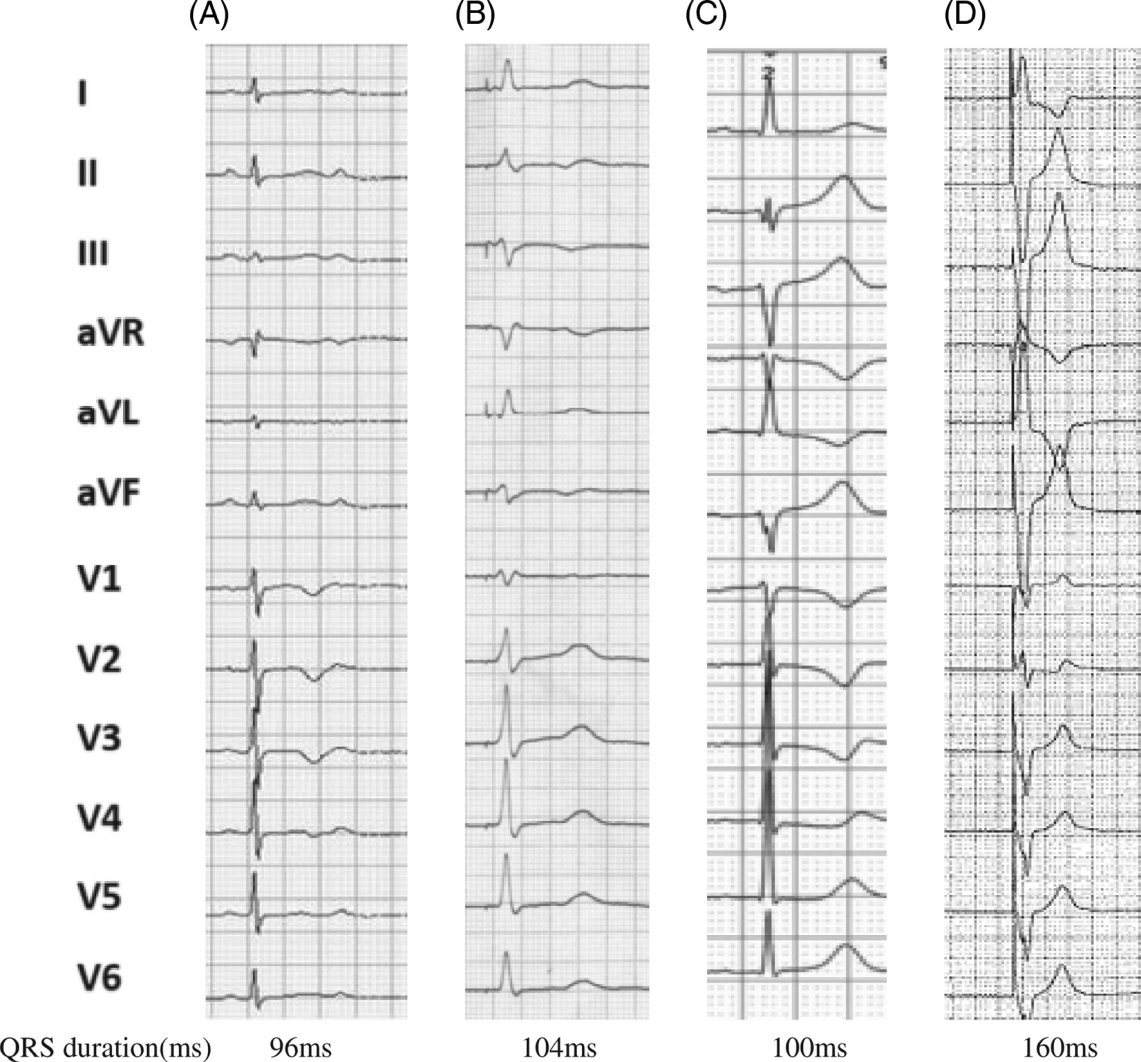
Comparison of QRS duration of LBBP and RVAP. (A) Intrinsic rhythm; (B) Left bundle branch pacing; (C) Intrinsic rhythm; (D) Right ventricular apical pacing

In 2017, Huang et al. described an case report, who was troubled by dilated cardiomyopathy (DCM) and left bundle branch block (LBBB), and treated with left bundle branch pacing (LBBP); found improvements in cardiac function (LVEF got higher, from 32% to 62%).^12^ The feasibility and safety of LBBP has subsequently been demonstrated by several studies. LBBP may be a new pacing strategy, on account of low threshold and narrow paced ECG QRS duration.^13^ The aim of this study was to evaluate the clinical outcomes of LBBP compared to RVAP (Figure 1).

## 2 METHODS

### 2.1 Study population

This was a single‐center, retrospective, observational study. We studied patients referred to Zhenjiang NO1 People's hospital, from January 2018 to December 2018 for permanent pacemaker implantation for standard indications.^14^ All patients were troubled by high‐degree atrial‐ventricular (AV) block or three‐degree AV block. The patients in this study were divided into two groups based on the pacing site. One group of patients received traditional RVAP (RVAP group) and the other group received LBBP pacing (LBBP group). All patients were >18 years of age; patients were excluded if they were younger than 18 years of age, underwent cardiac resynchronization therapy or had existing cardiac implantable devices. All patients have signed written informed consent agreeing to the implantation procedure, and the protocol was approved by the hospital Institutional Review Board.

### 2.2 Procedure

**FIGURE 2:**
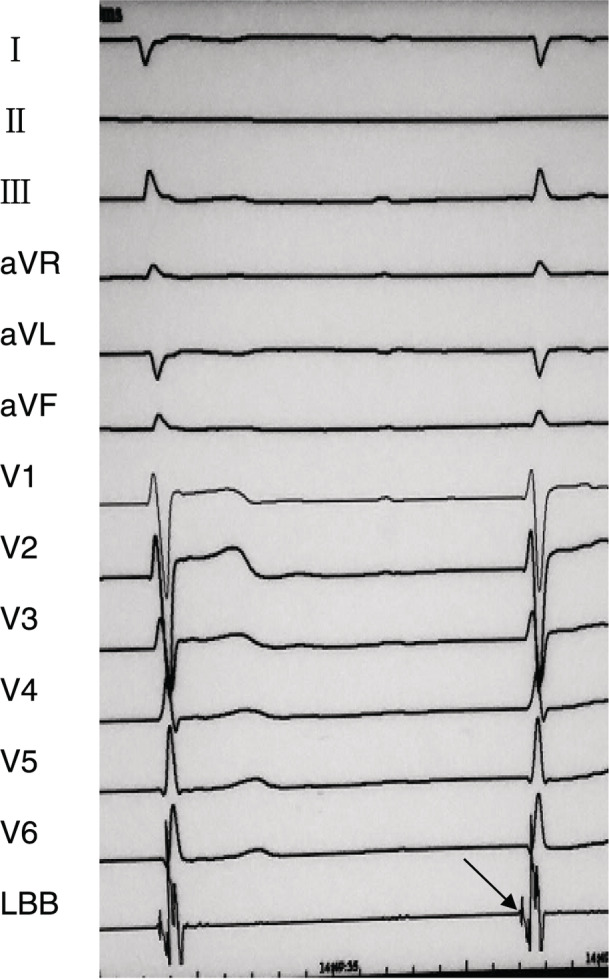
Left bundle branch potential. Left bundle branch potential (arrow) in intracardiac EGM during intrinsic rhythm

LBBP: The delivery sheath (C315 HIS, Medtronic Inc, Minneapolis, MN) was inserted into the right ventricle near the tricuspid annulus via the left subclavian vein or axillary vein. Then, the Select Secure pacing lead (3830, 69 cm, Medtronic Inc, Minneapolis, MN) was advanced through the sheath and placed in the His bundle region, with its distal electrode just beyond the tip of the sheath for unipolar pacing and local electric potential recording. The location of His bundle would help certify the insertion site for LBBP. Subsequently, the sheath with the pacing lead was further moved in the ventricular apex direction (about 1–1.5 cm) in right anterior oblique (RAO 30°) fluoroscopy view. The pacing lead was then screwed towards the left side of septum perpendicularly, while carefully monitoring the paced ECG morphology and pacing impedance. Once the paced ECG QRS morphology presented right bundle branch block (RBBB) pattern, further advancement of the lead was stopped. The pacing parameters were measured to confirm acceptable capture threshold, pacing impedance and sensing amplitude; then, the sheath was withdrawn. The stimulus to peak left ventricular activation time (S‐PLVAT), defined as the duration between the ventricular stimulation signal and R spike in lead V5, was also measured. Successful LBBP was characterized as a paced QRS morphology of RBBB pattern, and S‐PLVAT shortens abruptly with increasing output or remains shortest and constant at low and high outputs. In addition, if an LBB potential could be recorded during intrinsic rhythm, an indication of direct LBB pacing, the interval from the potential to the beginning of the QRS complex was measured. The left bundle branch potential (LBB potential) was observed in 58.6% cases (17/29) during intrinsic rhythm (Figure 2).

RVAP: The pacing lead (ICM09B,58 cm,Vitatron Holding B.V.) was positioned in the right ventricular apex in a standard fashion. In both groups, dual‐chamber pacemakers were implanted with the atrial pacing leads being implanted in the right atrial appendage.

### 2.3 Follow‐up

Patient demographics, medical history, electrocardiographic and echocardiographic findings were collected routinely. Electrocardiogram and echocardiography were performed by specialists in our hospital, LVEF is calculated by Simpson method. Pacing parameters(capture threshold, impedance, and sensing anplitude) were recorded at implant and during device follow‐ups. Patients were followed in the device clinic for a minimum of 12 months and up to 24 months at a 3–6 monthly interval.

HFH and new‐onset AF were tracked at follow‐ups. HFH was defined as an unplanned outpatient or emergency department visit or inpatient hospitalization in which the patient presented with symptoms and signs consistent with heart failure, evaluated by two independent cardiologists. New‐onset AF was obtained via pacemaker program controller, defined as AF that lasted more than 30 s.

### 2.4 Statistical analysis

Continuous variables were summarized as mean ± SD, categorical variables were summarized as number and percentages. Differences in mean values between two groups or two time points were compared using Student *t*‐test for continuous variables. The *χ* ^2^ test or Fisher's exact test (if the sample size was less than 40 or the minimum theoretical frequency was less than (1) were used for categorical variables. A two‐sided *p* value <.05 was considered statistically significant. All statistical analyses were performed using the Software SPSS 22.0 (SPSS Inc, Armonk, NY).

## 3 RESULTS

### 3.1 Baseline characteristics

During the study period, 70 patients with AVB underwent permanent pacemaker implantation and met the inclusion criteria. LBBP was attempted in 33 consecutive patients, among which the surgery was successful in 29 patient (87.9%), and the other 4 patients (12.1%) failed LBBP and underwent RVAP instead. The remaining 37 patients underwent RVAP as planned, and all surgeries were successful(100%). The mean age was 65.50 ± 8.79 years with males accounting for 58.6% of the study cohort. Prior history of heart failure and atrial fibrillation was present in 17.14% (n = 12) and 10.00% (n = 7), respectively. The mean follow‐up in the LBBP group was 17.40 ± 3.41 months compared to 18.00 ± 3.30 months (*p* = .69) in the RVAP group, and no patient was lost to follow up. Baseline demographics, pre‐implantation medical history, left ventricular ejection fraction and QRS width were similar between the two groups. Baseline characteristics are shown in Table 1.

**TABLE 1:** : Patient baseline characteristics

	LBBP group (N = 29)	RVAP group (N = 37)	*p*‐value
Age, mean (SD)	63.60 ± 8.80	67.40 ± 8.81	.347
Male, N (%)	13(44.83%)	17(45.95%)	.620
Hypertension, N (%)	17(58.62%)	16(43.24%)	.386
Diabetes,(N%)	9(31.03%)	6(16.22%)	.150
Coronary artery disease, N (%)	7(24.14%)	12(32.43%)	.292
Heart failure, N (%)	4(13.80%)	8(21.62%)	.324
Atrial fibrillation, N(%)	4(13.80%)	3(8.11%)	.677
QRS duration (ms), mean (SD)	104.83 ± 15.41	98.86 ± 7.33	.238
LVEF%, mean (SD)	55.08 ± 4.32	56.29 ± 5.40	.541
LVDD (mm), mean (SD)	48.71 ± 3.27	46.92 ± 4.931	.277

*Note:* Values are mean (SD), or number (%). *p*‐value<.05 was considered statistically significant.

Abbreviations: LBBP, left bundle branch pacing; LVDD, left ventricular end diastolic dimension; LVEF, left ventricular ejection fraction; RVAP, right ventricular apical pacing.

### 3.2 Implant outcomes

There were no significant differences in sensing amplitude, pacing impedance, and capture threshold between LBBP group and RVAP group at implantation and at last follow‐up (Table 2). Cum% VP was similar between the two groups (95.47 ± 1.22 vs. 94.86 ± 1.56, *p* = .768). Paced QRS duration was significantly narrower in the LBBP group compare to RAP (110.75 ± 6.77 vs. 154.29 ± 6.96, *p* = .000) at implantation (Figure 1), and the difference persisted during follow‐up. The LBB potential was recorded in 58.6% of LBBP patients, and the interval from LBB potential to the beginning of ECG QRS was 22.26 ± 4.32 ms. QRS duration was 108.84 ± 6.56 ms during LBBP in patients with recorded LBB potential during intrinsic rhythm and 113.67 ± 7.26 ms during LBBP in patients without recorded LBB potential (*p* = .386). At the end of follow‐up, we found the left ventricular end diastolic dimension (LVDD) was shorter and the LVEF% was higher in the LBBP group, significantly.

**TABLE 2:** Pacing, QRS duration, and echocardiographic characteristics

		LBBP group (N = 29)	RVAP group (N = 37)	*p*‐value
Measurements at implantation	Paced QRS duration (ms), mean (SD)	110.75 ± 6.77	154.29 ± 6.96	.000
	Capture threshold (V @0.4 ms), mean (SD)	0.68 ± 0.22	0.73 ± 0.23	.620
	R wave amplitude (mV), mean (SD)	9.42 ± 2.05	9.82 ± 2.01	.617
	Ventricular impedance (Ohms), mean (SD)	818.83 ± 165.73	844.29 ± 182.95	.715
Measurements at last follow‐up	Paced QRS duration (ms), mean (SD)	111.83 ± 6.89	155.36 ± 5.94	.000
	Capture threshold (V@0.4 ms), mean (SD)	0.65 ± 0.22	0.71 ± 0.21	.511
	R wave amplitude (mV), mean (SD)	10.25 ± 1.95	10.36 ± 1.80	.886
	Ventricular impedance (Ohms), mean (SD)	826.67 ± 164.45	856.71 ± 160.73	.643
	LVEF%, mean (SD)	54.17 ± 4.34	50.14 ± 2.14	.005
	LVDD (mm), mean (SD)	47.58 ± 3.29	49.79 ± 1.85	.042
	Cum% VP	95.47% ± 1.22%	94.86% ± 1.56%	.768

*Note:* Values are mean (SD), or number(%). *p*‐value<.05 was considered statistically significant.

Abbreviations: LBBP, left bundle branch pacing; LVDD, left ventricular end diastolic dimension; LVEF, left ventricular ejection fraction; RVAP, right ventricular apical pacing.

We compared the pacing parameters before and after surgery. As shown in Table 3,pacing parameters remained stable during follow‐up period, including the pacing threshold, sensing amplitude and impedance in two groups. In the LBBP group, the paced QRS duration was 111.83 ± 6.89 at last follow‐up, that was not different from that at implantation (110.75 ± 6.77, *p* = .684). In patients with LBBP, we observed a stable LVEF (55.08 ± 4.32 vs. 54.17 ± 4.34, *p* = .609) and LVDD (48.71 ± 3.27 vs. 47.58 ± 3.29, *p* = .700). On the contrary, in patients with RVAP, the LVEF got lower (56.29 ± 5.40 vs. 50.14 ± 2.14, *p* = .005) and the LVDD got longer (46.92 ± 4.93 vs. 49.79 ± 1.85, *p* = .046) during follow‐ups (Table 3).

**TABLE 3:** Changes in pacing parameters at follow‐ups

	LBBP group (N = 29)			RVAP group (N = 37)		
	At implantation	At last follow‐up	*p*‐value	At implantation	At last follow‐up	*p*‐value
Paced QRS duration(ms), mean(SD)	110.75 ± 6.77	111.83 ± 6.89	.684	154.29 ± 6.96	155.36 ± 5.94	.704
Capture threshold(V @0.4 ms), mean(SD)	0.68 ± 0.22	0.65 ± 0.22	.786	0.73 ± 0.23	0.71 ± 0.21	.837
R wave amplitude(mV), mean(SD)	9.42 ± 2.05	10.25 ± 1.95	.412	9.82 ± 2.01	10.36 ± 1.80	.440
Ventricular impedance(Ohms), mean(SD)	818.83 ± 165.73	826.67 ± 164.45	.772	844.29 ± 182.95	856.71 ± 160.73	.832
LVEF%, mean(SD)	55.08 ± 4.32	54.17 ± 4.34	.609	56.29 ± 5.40	50.14 ± 2.14	.005
LVDD(mm), mean(SD)	48.71 ± 3.27	47.58 ± 3.29	.700	46.92 ± 4.93	49.79 ± 1.85	.046

*Note:* Values are mean (SD), or number(%). *p*‐value<.05 was considered statistically significant.

Abbreviations: LBBP, left bundle branch pacing; LVDD, left ventricular end diastolic dimension; LVEF, left ventricular ejection fraction; RVAP, right ventricular apical pacing.

During the study period, there were 12 HFH events, there was a significant decrease in HFH in all patients with LBBP (2/29, 6.90%) compared to RAP (10/37, 27.03%; *p* = .024); there were 16 AF recorded, there was a significant decrease in LBBP group (4/29, 14.79%) compared to RVAP group (12/37, 32.43%; *p* = .046) .

## 4 DISCUSSION

The results of our study show that LBBP is associated with a significant reduction in HFH and AF in comparison with conventional RVAP in patients undergoing permanent pacemaker implantation. The LBBP group's LVEF and LVDD were stable at the end of follow‐up, and better than RVAP group, despite the high rate of ventricular pacing(Cum% VP = 95.47% ± 1.22%). The present study demonstrates that LBBP is a safe and effective physiological pacing procedure.

Ventricular desynchronization imposed by right ventricular pacing (RVP) increases the risk of HFH, AF, and mortality.^2^, ^3^, ^15^ Recently, Raghav Bansal et al. demonstrated that incidence of pacemaker‐induced cardiomyopathy (PiCMP) with RVP was found to be 13.8% over a mean follow‐up of 14.5 months. HBP is a relatively mature method for physiological pacing, and some researches indicates that HBP is associated with reduction in mortality and HFH compared to RVP. But there are shortcomings or challenges with HBP, such as a high capture threshold, lead dislocation rate, particularly in those with pathological disease in the conduction system.^8^, ^9^, ^10^, ^11^

The QRS duration has been accepted as a surrogate for the evaluation of electrical synchrony.^16^ The present study shows that the paced QRS duration was significantly shorter with LBBP compared with RVAP and did not prolonged compared with intrinsic QRS duration. During LBBP, the 3830 lead was rotated into the ventricular septum and fixed in the left bundle branch area, then the left ventricular His‐Purkinje system was paced directly, which results in a shorter paced QRS duration and better electrical synchrony. The LBB potential was recorded in 26.7% to 80%^17^, ^18^, ^19^, ^20^ of LBBP patients in recent studies, the percentage of recorded LBB potential was 58.6% (17/29).

In 2019, the research of Hou X et al. showed that left ventricular synchrony in the LBBP group was superior to that of the right ventricular septal group and similar to that in HBP group, measured by single‐photon emission computed tomography myocardial perfusion imaging.^17^ Binni Cai et al. assessed cardiac synchrony parameters using echocardiography in 78 patients with LBBP (n = 40) or right ventricular septal pacing (n = 38). LBBP maintained a good left ventricular mechanical synchrony that was similar to that of native conduction and was significantly better than that of right ventricular septal pacing. The mechanism for avoiding damage to cardiac function and preventing HFH and AF after pacemaker implantation in our study maybe due to the maintenance of ventricular synchrony during LBBP. In 2019, Li X et al. reported the similar results at 3 months follow‐up,^19^ but the follow‐up time in our study is much longer.

The present study has confirmed the safety and feasibility of LBBP with a success rate of 87.9%, which is identical to previous studies showing the LBBP success rate is 80.5% to 92.4%.^20^, ^21^ The surgery failed in four patients, the reasons were as follows: in two cases, when removing the C315 sheath, the 3830 lead was pulled which dislocated the lead. In the third case, the lead tip was damaged by repeated procedural maneuvers. In another case, there was an anterior myocardial infarction, the 3830 lead could not be screwed at multiple locations potentially because of severe fibrosis.

This study demonstrated that the capture threshold with LBBP was low and stable, with no significant difference as compared to RVAP. LBBP is achieved by trans‐ventricular‐septal method and the pacing lead is positioned in the basal ventricular septum. Left bundle branch usually spreads below the membranous atrioventricular septum with a large dimension and there is less fibrosis wrapped than His bundle.^22^ Thus, it is not surprising that a low capture threshold was noted. Meanwhile, R‐wave amplitude with LBBP was high and stable, which guarantees appropriate sensing.

As a novel pacing strategy, how to define the concept of LBBP is still unknown. There are several findings in our study to help confirm LBBP. (i) the pacing lead tip was confirmed beneath the endocardium of the left ventricular septum; (ii) twelve‐lead ECG showed the pattern of right bundle branch block;(iii) S‐PLVAT shortens abruptly with increasing output or remains shortest and constant at low and high outputs. Recently, Hou x et al. showed that left ventricular septal pacing plus the record of LBB potential might generate the new concept of LBBP, but the mechanisms are unknown.^17^

Complications regarding LBBP should be noted except for conventional complications of transvenous pacing. Though none was observed in our study, complications like lead perforation, ventricular septal coronary damage and lead fracture should be taken seriously.

LBBP is a feasible, safety and most important, physiological pacing procedure. Our study provides basis for its widespread clinical use. We believe the study will offer some help for further researches.

### 4.1 Study limitations

Several limitations should be mentioned. First, this was a retrospective and observational study in a single centre, therefore, the results may be not representative. Second, the definition, evaluation standard and operating procedure of LBBP have not been normalized and unified, the success rate and outcomes may not be exactly the same. Large, prospective, randomized trials are necessary to evaluate the procedure's safety, and to prove mortality and heart failure benefits attributable to LBBP.

## 5 CONCLUSIONS

The present investigation demonstrates the safety and feasibility of LBBP that produces narrower paced QRS duration than RVAP. The LBBP low capture threshold and high R‐wave sensing amplitude favor long‐term pacing management and device longevity. LBBP is associated with reduction in the occurrence of pacing‐induced left ventricular dysfunction and HFH compared to RVAP in patients requiring permanent pacemakers.

## CONFLICT OF INTEREST

The authors declare no potential conflict of interest.

## Data Availability

All data, models, and code generated or used during the study appear in the submitted article.
